# CFH Loss in Human RPE Cells Leads to Inflammation and Complement System Dysregulation via the NF-κB Pathway

**DOI:** 10.3390/ijms22168727

**Published:** 2021-08-13

**Authors:** Angela Armento, Tiziana L. Schmidt, Inga Sonntag, David A. Merle, Mohamed Ali Jarboui, Ellen Kilger, Simon J. Clark, Marius Ueffing

**Affiliations:** 1 Institute for Ophthalmic Research, Department for Ophthalmology, Eberhard Karls University of Tübingen, 72076 Tübingen, Germany; TizianaLuisa@web.de (T.L.S.); inga.sonntag@uni-tuebingen.de (I.S.); david.merle@medunigraz.at (D.A.M.); mohamed-ali.jarboui@uni-tuebingen.de (M.A.J.); ellen.kilger@uni-tuebingen.de (E.K.); simon.clark@uni-tuebingen.de (S.J.C.); 2 Department of Ophthalmology, Medical University of Graz, 8036 Graz, Austria; 3 Department for Ophthalmology, University Eye Clinic, Eberhard Karls University of Tübingen, 72076 Tübingen, Germany; 4 Lydia Becker Institute of Immunology and Inflammation, Faculty of Biology, Medicine and Health, University of Manchester, Manchester M13 9PT, UK

**Keywords:** age-related macular degeneration (AMD), retinal pigment epithelium (RPE) cells, complement factor H (CFH), inflammation, cytokines, NF-κB pathway

## Abstract

Age-related macular degeneration (AMD), the leading cause of vision loss in the elderly, is a degenerative disease of the macula, where retinal pigment epithelium (RPE) cells are damaged in the early stages of the disease, and chronic inflammatory processes may be involved. Besides aging and lifestyle factors as drivers of AMD, a strong genetic association to AMD is found in genes of the complement system, with a single polymorphism in the complement factor H gene (*CFH*), accounting for the majority of AMD risk. However, the exact mechanism of *CFH* dysregulation confers such a great risk for AMD and its role in RPE cell homeostasis is unclear. To explore the role of endogenous *CFH* locally in RPE cells, we silenced *CFH* in human hTERT-RPE1 cells. We demonstrate that endogenously expressed *CFH* in RPE cells modulates inflammatory cytokine production and complement regulation, independent of external complement sources, or stressors. We show that loss of the factor H protein (FH) results in increased levels of inflammatory mediators (e.g., IL-6, IL-8, GM-CSF) and altered levels of complement proteins (e.g., *C3, CFB* upregulation, and *C5* downregulation) that are known to play a role in AMD. Moreover, our results identify the NF-κB pathway as the major pathway involved in regulating these inflammatory and complement factors. Our findings suggest that in RPE cells, FH and the NF-κB pathway work in synergy to maintain inflammatory and complement balance, and in case either one of them is dysregulated, the RPE microenvironment changes towards a proinflammatory AMD-like phenotype.

## 1. Introduction

The specific anatomic structure of the human eye permits a tightly regulated local immune response to be sufficient in protecting the retina from external pathogens and to maintain its visual function [1]. In immune-privileged organs, like the eye, an excessive immune response, and the subsequent recruitment of circulating immune cells, may lead to tissue damage and affect the function of such a highly specified organ. Ocular immune-privilege is mainly ensured by the physical barrier formed by the retinal pigment epithelium (RPE) cell monolayer sitting on an extracellular matrix (ECM), called Bruch’s membrane (BrM), which separates the neurosensory retina from the choroid and choriocapillaris [2]. The main advantage of an intact RPE/BrM interface is that it provides an effective barrier for the selectivity of molecular diffusion, especially with regard to a possible systemic inflammatory insult [3]. Considering that the composition of BrM relies on the deposition of ECM components from both the endothelium cells of the choriocapillaris and the RPE cells, any disruption to RPE cell homeostasis is deleterious for effective barrier maintenance. Moreover, RPE cells exert several other functions needed for retinal health. RPE cells are not only responsible for the phagocytosis and recycling of photoreceptor outer segments (POS), but they also possess antioxidant activity and actively take up nutrients from, and release discard material into the BrM [4]. Although increased signs of inflammation are observed in several retinal degenerative diseases [5], the combination of RPE cell dysfunction, barrier breakdown, and subtle, chronic, inflammation are characteristic for the disease age-related macular degeneration (AMD) [6].

AMD is a progressive degenerative disease of the retina, which leads to patients losing their central vision, and in later stages, suffering blindness [7]. AMD affects foremost the elderly population, and it is estimated that with increasing life expectancy, around 300 million people will be affected by 2040 [8]. A hallmark of the disease is the presence of deposits, called drusen, within BrM underneath the RPE cells, which not only impair RPE function, but also greatly alter the properties of BrM [9]. The events that lead to these changes are not yet fully understood; however, it is known that AMD is caused by a combination of aging, genetic predisposition, and lifestyle [10,11,12]. The majority of genetic risk lies in the genes of the alternative pathway of the complement system [13], which is an important part of the innate immune system. The canonical role of the complement system is to recognize and mediate the removal of pathogens, debris, and dead cells via the activation of the complement proteolytic cascade [14]. Clearly, tight regulation of complement activation is required to prevent inflammation-induced tissue damage, especially in an immune-privileged organ like the eye [15]. At the site of complement activation, the release of the cleaved complement factors C3a and C5a, called anaphylatoxins, leads to the recruitment and activation of circulating immune cells, such as macrophages and leucocytes [16]. Additionally, C3a and C5a activate resident immune cells, like microglia and Muller cells, generating a chronic inflammatory environment, which is observed in AMD [17,18]. Complement dysregulation is not only linked to AMD via genetic association. Several complement activation products have also been detected in drusen, as well as in the eyes and in the blood of AMD patients [9,19,20]. One of the most common genetic risks, accounting for 50% of attributable risk for AMD, corresponds to a polymorphism in the complement factor H (*CFH*) gene that consists of a Tyr to His amino acid substitution at position 402 in the preprocessed factor H protein (FH: Position 384 in the mature FH protein) [21,22]. The Y402H polymorphism is also present in the alternative splicing product of the *CFH* gene, the protein called factor H-like protein 1 (FHL-1), which is around a third of the size of FH and found to predominate in BrM [23]. FH and FHL-1 are negative regulators of the alternative pathway of the complement system and promote the degradation of C3b, a breakdown product of C3 and the central component of the complement activation amplification loop [24]. The AMD high-risk genetic variant *CFH* 402H is believed to be involved in AMD pathogenesis in different ways. Indeed, besides the fact that the FH/FHL-1 402H variant has been associated with increased complement activation [25], the same variant also shows reduced binding affinity to ECM components (e.g., heparan sulfate) [26], oxidized lipids (e.g., malondialdehyde MDA) [27] and inflammatory mediators (e.g., C-reactive protein CRP) [28,29]. Most importantly, the function of the FH/FHL-1 proteins may differ depending on their source and location [24,30]. Indeed, in this regard, the endogenous impact of *CFH* proteins in RPE cells has rarely been investigated. In our recent study, we unraveled a non-canonical function of endogenous FH, as the predominant splice form found in RPE cells. By silencing *CFH* in hTERT-RPE1 cells, we showed that FH loss in RPE cells not only modulates the extracellular microenvironment via its regulation of C3 levels, but also has an intracellular impact on the antioxidant functions and metabolic homeostasis of RPE cells [31]. In the current study, the same model was employed to investigate the endogenous role of FH in the inflammatory response of RPE cells, since RPE cells actively contribute to the maintenance of the immune-privileged status of the eye, and not only via their barrier function. In particular, we focused on the interactions between FH, inflammation, and the nuclear factor kappa-light-chain-enhancer of activated B cells (NF-κB) pathway in RPE cells. The NF-κB pathway is a known key regulator of inflammation. Upon canonical regulation of this pathway, the p65 subunit (RelA) of the NF-κB complex is phosphorylated and translocates to the nucleus, where it promotes the transcription of several NF-κB target genes, including inflammatory cytokines, chemokines and also genes involved in oxidative stress response [32]. Activation of the NF-κB pathway has been associated with several neurodegenerative diseases, including Alzheimer’s and Parkinson’s disease [33], and retinal degenerative diseases, such as diabetic retinopathy [34].

Here, we show that RPE cells are immunocompetent with respect to their ability to express and regulate immune-modulatory genes, including cytokines and chemokines. FH loss results in an increase of inflammatory cytokines and chemokines in an NF-κB dependent fashion. Moreover, we discovered that FH loss strongly alters the regulation of other complement genes, again in an NF-κB dependent way, thereby creating dysfunction in complement pathway regulation. As such, the NF-κB pathway emerged as a major signaling pathway controlling immune competence and response in RPE cells.

## 2. Results

### 2.1. CFH Loss Leads to Upregulation of C3 and Inflammatory Cytokines in RPE Cells

In our previous study, we used siRNA silencing of the *CFH* gene to investigate the impact of reduced FH levels and activity in hTERT-RPE1 cells in response to oxidative stress after 48 h in vitro [31]. Here, we used the same model to investigate the immune reactivity of RPE cells after *CFH* knockdown and prolonged its silencing period up to six days (144 h). Using both the short (48 h) and the prolonged (144 h) silencing period, we investigated the impact of endogenous FH loss on proinflammatory cytokine production and complement system regulation.

**Figure 1:**
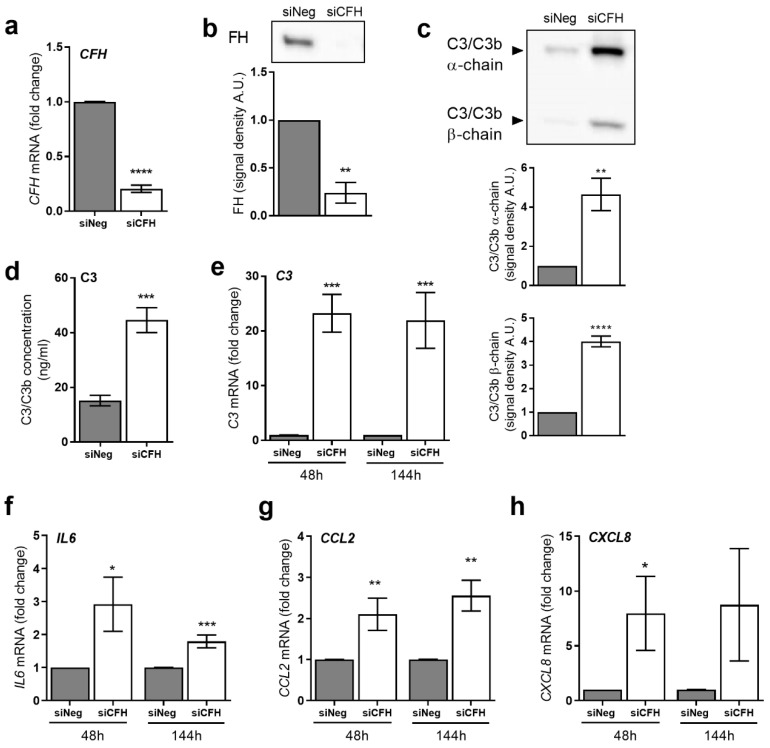
Short and sustained FH reduction leads to increased expression levels of C3 and inflammatory cytokines. (**a**) Evaluation of *CFH* expression after 144 h by qRT-PCR analyses. Data are normalized to the housekeeping gene *PRLP0* using Δ ΔCt methods. SEM is shown, n = 5. (**b**) Western blot analyses of FH protein levels in cell culture supernatants of hTERT-RPE1 cells after 144 h. Quantification of the signal density of three independent experiments is shown. (**c**) Western blot analyses of C3/C3b α-chain and β-chain protein levels in cell culture supernatants of hTERT-RPE1 cells after 144 h. Quantification of the signal density of five independent experiments is shown. (**d**) C3/C3b ELISA analyses of cell culture supernatants of hTERT-RPE1 cells after 144 h. SEM is shown, n = 6. (**e,h**) Monitoring of gene expression by qRT-PCR analyses in hTERT-RPE1 cells: (**e**) Complement component 3 (*C3*), (**f**) interleukin-6 (*IL6*), (**g**) C-C Motif Chemokine Ligand 2 (*CCL2*) and (**h**) interleukin-8 (*CXCL8*). Data are normalized to housekeeping gene *PRPL0* using Δ ΔCt method. SEM is shown, n = 5−8. Western blot images were cropped, and full-length blots are provided in Supplementary Material. Significance was assessed by a Student’s *t*-test. *p < 0.05, ** *p* < 0.01, *** *p* < 0.001, **** *p* < 0.0001. negative control (siNeg), *CFH* specific siRNA (siCFH).

Efficient *CFH* silencing after 48 h was already shown before and reproduced in this study [31]. Here, we show that also after 144 h, *CFH* mRNA was significantly reduced in *CFH* knockdown (siCFH), RPE cells compared to control cells (siNeg). The silencing efficiency of almost 90% after 48 h was also maintained at 144 h (Figure 1a). Likewise, FH protein levels remained almost undetectable in cell culture supernatants of siCFH cells after 144 h (Figure 1b). With prolonged FH loss, *C3* gene expression (Figure 1e) and C3 protein levels (Figure 1c,d) increased significantly. Levels of *C3* mRNA were found to be 20-fold higher in siCFH cells at both time points (Figure 1e). Similarly, protein levels of secreted C3 were significantly elevated in siCFH cells, as shown by a 2-fold increase in C3/C3b ELISA (Figure 1c) and a 4-fold increase in C3/C3b alpha and beta chains in Western blot (Figure 1d). Consistently, *CFH* silencing was performed on ARPE19 cells, and a comparable effect on C3 levels was observed, both at RNA and protein level (Figure S1a–c).

Given that early AMD is hallmarked by persistent inflammation [35,36], we investigated if FH might influence the levels of relevant inflammatory cytokines, including interleukin-6 (*IL6*), C-C Motif Chemokine Ligand 2 (*CCL2*), and interleukin-8 (*CXCL8*). When FH was downregulated in hTERT-RPE1 siCFH cells, we observed an upregulation of *IL6* (3-fold at 48 h and 2-fold at 144 h) and *CCL2* (2-fold at 48 and 2.5-fold at 144 h) (Figure 1f–g). Moreover, *CXCL8* levels were 8-fold upregulated in siCFH cells, significantly after 48h (Figure 1h). Likewise, *CFH* silencing in ARPE19 also led to increased gene expression levels of *IL6*, *CXCL8*, and *CCL2* (Figure S1d,f)

This indicates that reducing FH levels and activity in RPE cells leads to an upregulation of relevant proinflammatory molecules.

### 2.2. Cytokine Expression Mediated by FH Loss Is Driven by NF-κB Activity

**Figure 2:**
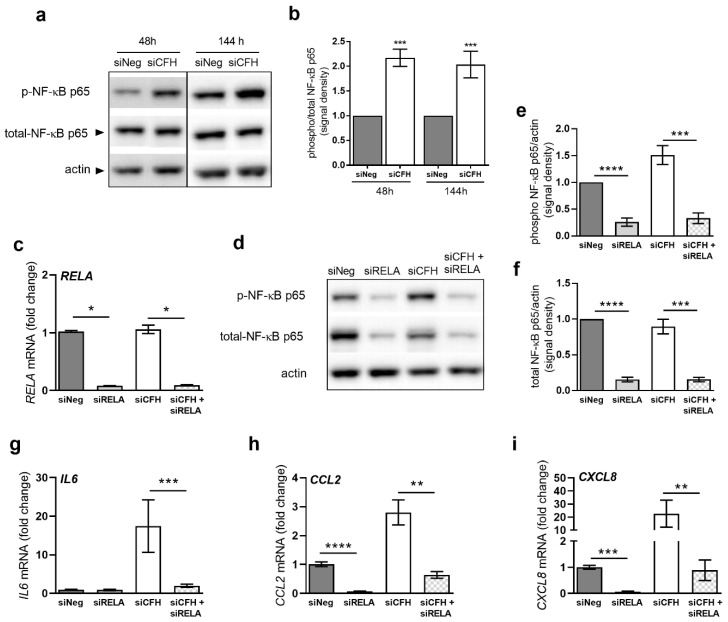
RPE cells deprived of FH show NF-κB activation, and blocking NF-κB abolishes the effects of FH loss on cytokine gene expression levels. a Western blot analyses of phosphorylated and total levels of NF-κB p65 subunit in cell lysates of hTERT-RPE1 cells after 48h and144h. Total actin was used as a loading control. (**b**) Quantification of the signal density, normalized to actin, of at least four independent experiments as reported in (**a**) Bars indicate the signal density ratio between levels of phosphorylated and total NF-κB p65 subunit. (**c**) Evaluation of *RELA* gene expression levels by qRT-PCR analyses in hTERT-RPE1 cells after 48h. Data are normalized to the housekeeping gene *PRPL0* using Δ ΔCt methods. SEM is shown, n = 5. (**d**) Western blot analyses of phosphorylated and total levels of NF-κB p65 subunit in cell lysates of hTERT-RPE1 cells after 48 h. Total actin was used as a loading control. (**e**) Quantification of the signal density of three independent experiments in the conditions reported in (**c**,**d**). Bars indicate the signal density ratio between the phosphorylated NF-κB p65 subunit and actin. (**f**) Quantification of the signal density of three independent experiments in the conditions reported in c-d. Bars indicate the signal density ratio between the total NF-κB p65 subunit and actin. (**g**,**h**,**i**) Gene expression analyses by qRT-PCR of hTERT-RPE1 cells in the conditions reported in c-d: (**g**) interleukin-6 (*IL6*), (**h**) C-C Motif Chemokine Ligand 2 (*CCL2*), and (**i**) interleukin-8 (*CXCL8*). Data are normalized to housekeeping gene *PRLP0* using Δ ΔCt method. SEM is shown, n = 3−5. Western blot images were cropped, and full-length blots are provided in Supplementary Material. Significance was assessed by a Student’s *t*-test. * *p* < 0.05, ** *p* < 0.01, *** *p* < 0.001, **** *p* < 0.0001. negative control (siNeg), *CFH* specific siRNA (siCFH), *RELA* specific siRNA (siRELA).

Changes in RPE gene expression after FH reduction, suggest that a proinflammatory pathway may be regulated by FH in RPE cells. As NF-κB plays a major role in regulating various cytokine expression levels [37], we next investigated if *CFH* silencing changed the activity of NF-κB. To do so, we monitored the levels of the activated phosphorylated form of the NF-κB p65 subunit and its total levels in siNeg and siCFH hTERT-RPE1 cells over time (Figure 2a). Experiments were also repeated in ARPE-19 cells (Figure S2a). We found a sustained increase in NF-κB activation levels, as shown by the increased ratio of phosphorylated/total NF-κB p65 (Figure 2b and Figure S2b).

To investigate a potential direct correlation between reduced FH activity and the observed NF-κB pathway activation, we concomitantly double silenced *CFH* and *RELA*, the gene coding for the NF-κB p65 subunit. In hTERT-RPE1 cells, *RELA* silencing efficiently reduced RelA gene expression by about 90% in siRELA and siCFH + siRELA treated cells. In the parallel, also protein levels of NF-κB p65 were greatly reduced (Figure 2d). *CFH* silencing alone had no impact on *RELA* mRNA or p65 protein expression (Figure 2c,d). Quantification of protein levels for both the phosphorylated form of NF-κB p65 (Figure 2e) and total NF-κB p65 (Figure 2f) in siRELA and siCFH + siRELA treated cells show a significant reduction of protein abundance, confirming that silencing of *RELA* could effectively reduce NF-κB activation.

Next, we evaluated gene expression levels of the identified upregulated cytokines in response to RELA silencing. Interestingly, we found that under control conditions (i.e., in the presence of FH), the NF-κB pathway regulates the expression of *CCL2* and *CXCL8*, but not that of *IL6* (Figure 2g–i). As shown in Figure 2g, *IL6* levels only rise in the absence of FH activity (siCFH). Conversely, downregulation of NF-κB p65 in siCFH cells (siCFH + siRELA), lowers the gene expression of all three cytokines back to basal levels (Figure 2g–i). In particular, a strong significant reduction was observed for *IL6* (Figure 2g), *CXCL8* (Figure 2i), and for *CCL2* (Figure 2h).

Next, we tested whether exogenous application of FH could revert the effects of endogenous siRNA-based *CFH* suppression on both NF-κB activation, as well as the expression of inflammatory cytokines. At the same time, we evaluated the effects of the addition of C3 and C3b. The addition of exogenous complement factors, however, did not change NF-κB activation levels (Figure S3a,b) nor gene expression levels of *IL6* (Figure S3c) and *CCL2* (Figure S3d) at any of the time points tested (48 h and 144 h).

**Figure 3:**
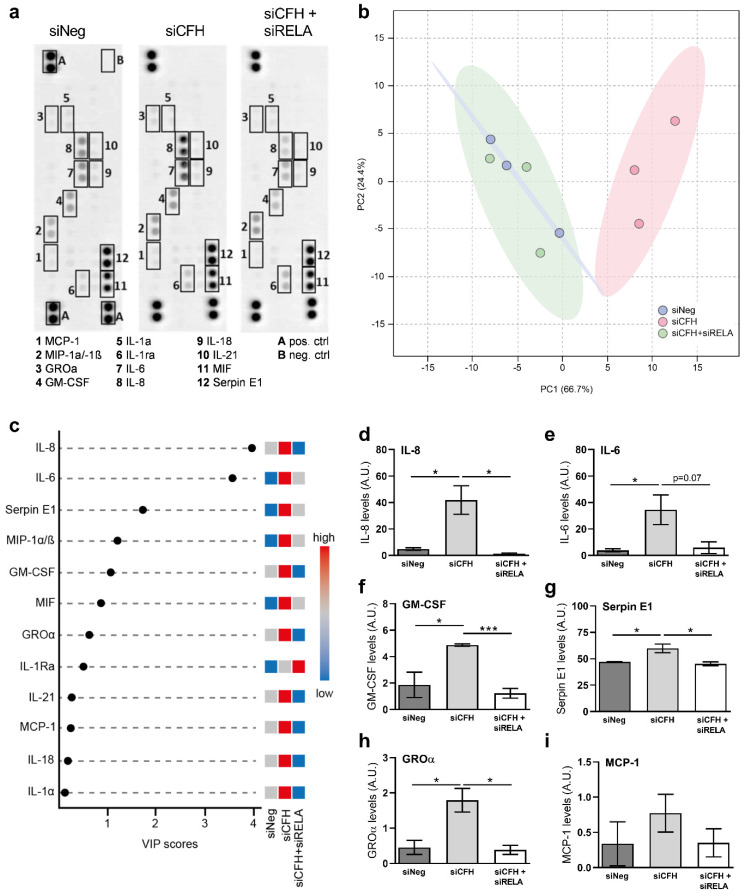
Blockade of NF-κB abolishes the effects of FH loss on secreted cytokines. (**a**) Representative image of a Proteome Profiler Human Cytokine Array analysis performed on cell culture supernatants collected from hTERT-RPE1 cells after 48 h. (**b**) PCA Analysis of the Cytokine array data for all biological replicates, samples were colored according to the corresponding siRNA treatment group, 95% confidence regions were plotted and colored according to each group. (**c**) Variable importance in projection (VIP) score plot derived from PLS-DA analysis, the top cytokines that contribute to the segregation between the groups were plotted, and their differential abundance was color scaled according to their enrichment (red), depletion (blue), or unchanged (grey). (**d**,**i**) Quantification of the signal density in the conditions reported in a: (**d**) Interleukin-8, IL-8; (**e**) interleukin-6, IL-6; (**f**) colony stimulating factor 2, GM-CSF; (**g**) serpin family E member 1, Serpin E1; (**h**) C-X-C Motif Chemokine Ligand 1, CXCL1/GROα; **i** C-C Motif Chemokine Ligand 2,CCL2. SEM is shown. n = 3. Significance was assessed by a Student’s *t*-test. * *p* < 0.05, *** *p* < 0.001). negative control (siNeg), *CFH* specific siRNA (siCFH), *RELA* specific siRNA (siRELA).

To assess whether the changes in gene transcription would translate into an inflammatory microenvironment outside of the RPE cells, we monitored the levels of secreted inflammatory factors via a cytokine array analyzing the serum-free conditioned medium supernatant of hTERT-RPE1 cells after 48 h (Figure 3a).

Using PCA analysis, we plotted all the signal intensities for each cytokine from all the biological replicates of each group: siNeg, siCFH, and siCFH+siRELA. As shown in Figure 3b, there is clear segregation between siNeg and siCFH as they cluster apart according to the first principal component (PC1), thus indicating a clear effect of siCFH silencing on the RPE cytokine signature. The combination of siCFH + siRELA had little effect on the cytokine profile signature as siCFH + siRELA group cluster tightly with siNeg control (Figure 3b). This suggests that downregulation of NF-κB p65 (siRELA) in siCFH silenced RPE cells results in a reversion of the proinflammatory phenotype to normal para-inflammatory homeostasis. As the microarray used for analysis only covers a limited number of cytokines, and their relative quantification was based on differences in signal detection after blotting, we chose to use a more supervised approach. Variable Importance in Projection (VIP) values from Partial Least Square Discriminant Analysis (PLS-DA) was used to gain a quantitative estimation of the discriminatory power of each individual cytokine (Figure 3c). VIP score analysis detected 12 cytokines that significantly differentiated between the three siRNA groups. IL-8 and IL-6, also shown in Figure 3d,e are the two cytokines that contribute the most to the segregation between siNeg, siCFH, and siCFH + siRELA. Besides these two, most of the cytokines analyzed on the array were increased in the siCFH group when compared to the siNeg controls: Colony-stimulating factor 2 (GM-CSF, Figure 3f), serpin family E member 1 (Serpin E1, Figure 3g), C-X-C Motif Chemokine Ligand 1 (CXCL1/GROα, Figure 3h), C-C motif chemokine ligand 3 and 4 (MIP-1α/-1β, Figure S4a), while the effects on MCP-1, IL18 and IL-1a were less pronounced and only slightly changed (Figure 3i). Most of these cytokines exhibit a decreased level of abundance when silencing of FH (siCFH), and NF-ƙB p65 (siRELA) was combined: IL-8 (Figure 3d), IL-6 (Figure 3e), GM-CSF (Figure 3f), Serpin E1 (Figure 3g), CXCL1/GROα were reduced to a base level indicating that inhibition of the NF-κB pathway can dampen or abrogate the consequences of FH loss (Figure 3h).

An exception to this pattern was seen with interleukin 1 receptor antagonist (IL-1Ra, (Figure 3a,c, and Figure S4b), which antagonizes the inflammatory effects of IL-1α/-1β via competitive binding to their receptors. The upregulation of IL-1Ra as an anti-inflammatory cytokine suggests that its expression is negatively regulated by NF-κB pathway activity. Minimal differences were observed in between the conditions (Figure S4) for macrophage migration inhibitory factor (MIF), interleukin-1 alpha (IL-1a), and interleukin-18 (IL-18) and interleukin-21 (IL-21), although the latter was slightly reduced in response to siRELA.

### 2.3. FH Loss Alters Transcription of Complement Genes via the NF-κB Pathway

The transcriptional regulation of complement genes has remained poorly understood in RPE cells, as well as in general. After finding that reduction of FH activity resulted in a marked upregulation of *C3* expression (Figure 1), we investigated whether either suppression of FH expression or NF-κB activity would regulate the expression levels of additional complement factors or regulators.

**Figure 4:**
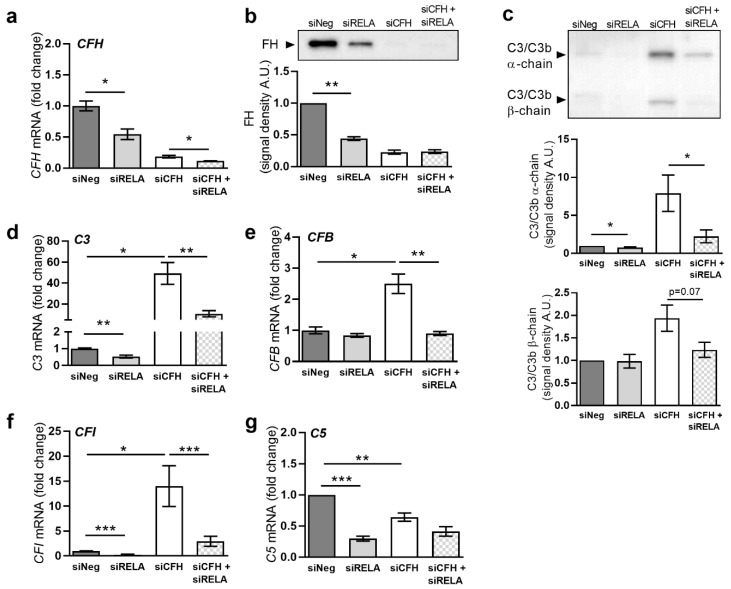
FH loss alters gene transcription of complement system genes via the NF-κB pathway. (**a**) Evaluation of *CFH* expression by qRT-PCR analyses. Data are normalized to the housekeeping gene PRPL0 using Δ ΔCt methods. SEM is shown, n = 5. (**b**) Evaluation of *C3* expression by qRT-PCR analyses. Data are normalized to the housekeeping gene PRPL0 using Δ ΔCt methods. SEM is shown, n = 5. (**c**) Western blot analyses of C3 α-chain and β-chain protein levels in cell culture supernatants. Quantification of the signal density of four independent experiments is shown. (**d**) Western blot analyses of FH protein levels in cell culture supernatants. Quantification of the signal density of three independent experiments is shown. (**e**) Evaluation of *CFB* expression by qRT-PCR analyses. (**f**) Evaluation of *CFI* expression by qRT-PCR analyses. (**g**) Evaluation of *C5* expression by qRT-PCR analyses. Data are normalized to the housekeeping gene PRPL0 using Δ ΔCt methods. SEM is shown, n = 5. Western blot images were cropped, and full-length blots are provided in Supplementary Material. Significance was assessed by a Student’s *t*-test, where * *p* < 0.05, ** *p* < 0.01, *** *p* < 0.001. Abbreviations used include: negative control (siNeg), *CFH* specific siRNA (siCFH), *RELA* specific siRNA (siRELA).

We observed a significant reduction of *CFH* mRNA levels in siRELA cells (Figure 4a), followed by reduced levels of FH protein (Figure 4b). C3 levels were also reduced in siRELA cells, both at the protein level (Figure 4c), as well as RNA level (Figure 4d). The addition of exogenous FH could only partially revert the effects of endogenous *CFH* silencing on levels of *C3* mRNA (Figure S5a) and secreted C3 (Figure S5b) after 144 h of incubation. Supplementation with C3 and C3b had no impact at any time point (Figure S5a).

Subsequently, we investigated gene expression levels of *CFB*, an important positive regulator of the alternative pathway of the complement system. We found a significant 2.5-fold increase in *CFB* gene expression after *CFH* silencing and a return to basal levels after silencing NF-κB p65, while *CFB* mRNA levels were not affected by siRELA alone (Figure 4e). The addition of exogenous FH could only partially revert the effects of endogenous *CFH* silencing on *CFB* mRNA levels, while addition of C3 and C3b had no effects (Figure S5c). Next, we evaluated the expression of *CFI*, a negative regulator of complement activation in the presence of FH as a co-factor. *CFI* mRNA levels were 12-fold higher in siCFH cells and were significantly reduced in siRELA cells (Figure 4f). Furthermore, we analyzed the expression of *C5*, another major component in the complement cascade. The levels of *C5* mRNA (Figure 4g) were reduced by both silencing FH expression in siCFH cells, as well as after NF-κB p65 silencing in siRELA cells.

Most importantly, for all complement system factors with increased expression in the absence of FH, levels were reduced via suppression of NF-κB activity. Thus, *CFB* mRNA levels were redirected to basal levels (Figure 4e), and mRNA levels of *CFI* were found clearly reduced (Figure 4f). *C3* mRNA levels were significantly reduced by half (Figure 4b), and levels of secreted C3 protein were also significantly reduced (Figure 4c).

## 3. Discussion

Given the strong association of the *CFH* gene with AMD, and a clear role of RPE cells in maintaining homeostasis in the retinal microenvironment, we investigated the role of FH in RPE cells with respect to its impact on balancing molecular mechanisms of inflammation. Here, we demonstrate that endogenously expressed *CFH* in RPE cells modulates inflammatory cytokine production and complement regulation, independent of external complement sources of stressors. We show that decreased *CFH* levels and activity result in increased levels of inflammatory cytokines, chemokines, and growth factors.

Although the RPE cell lines used in this study do not fully replicate the mature state of RPE cells, the phenotype caused by FH loss in this model, is strikingly similar to that of more mature RPE cells carrying an AMD-related *CFH* polymorphism or to the more physiological *cfh^−/−^* knockout mouse model.

Indeed, *cfh^−/−^* mice, which show signs of retinal degeneration similar to AMD pathology, present higher levels of complement activation and inflammation [38]. Interestingly, in the *cfh^−/−-^* mouse model, also other organs are pathologically affected by excessive inflammation and immune cell infiltration, such as the kidney [39] and the liver [40]. For example, *cfh^−/−^* mice face a spontaneous appearance of hepatocellular carcinomas with a clear transcriptomic signature for cytokine and chemokine signaling pathways [41]. Similarly, the phenotype of mature iPSC-RPE cells carrying the *CFH* Y402H AMD high-risk variant is characterized by the upregulation of several inflammatory cytokines, as IL6 [42,43] and C3b deposition [44].

In our model, the plethora of factors elevated in RPE cells upon FH dysregulation, are known to play a role in AMD, as well as in aging processes. Specifically, IL-6 and IL-8, which are the main discriminatory factors that allow clear segregation between control RPE cells (siNeg) and RPE cells deprived of *CFH,* are both members of the senescence-associated secretory pathway (SASP) and involved in aging processes. H_2_O_2_-mediated senescence in ARPE19 cells led to increased levels of IL-6 and IL-8 when FH levels were reduced [45]. Moreover, increased systemic IL-6 levels were found in patients with AMD, mostly in relation to the late subtypes of the disease [46]. Interestingly, a study exploring potential new drug targets for AMD identified IL-6 as a candidate target [47].

We found in RPE cells lacking FH increased secreted levels of GM-CSF (Figure 3), a growth factor that promotes activation and survival of microglia cells and macrophages [48]. Interestingly, elevated GM-CSF levels have also been found in the vitreous of postmortem human eyes genotyped for the *CFH* Y402H SNP. In the parallel accumulation of choroidal macrophages was observed [49]. Moreover, increased expression of GM-CSF was found after stimulation with the anaphylatoxins C3a and C5a in ARPE-19 cells [49]. Our data substantiate these findings, suggesting that RPE cells may be a source of GM-CSF found in the *CFH* Y402H postmortem eye.

Serpin E1, also known as Plasminogen Activator Inhibitor-1 (PAI-1), was upregulated in siCFH RPE cells. Serpin E1 is involved in ECM remodeling and angiogenesis [50], processes that are altered at the Bruch’s membrane/choroid interface in AMD [2]. Serpin E1 is also considered a senescence marker in several tissues [50]. High levels of Serpin E1 have been associated with neovascularization in AMD and diabetic retinopathy [51]. Serpin E1 mediates some of its effects via binding to the α5β3 integrin [52], and interestingly also FH and its truncated form FHL-1 modulate RPE function via binding a closely related integrin, α5β1 [53].

Other factors that were altered by FH in RPE cells in our study include CXCL1/GROα, a chemokine responsible for neutrophil recruitment and activation [54] that has been found increased in aqueous humor of AMD patients [55]; and MIP-1α, as well as MIP-1β, factors involved in inflammation-mediated damage in the retina [56]. FH loss in RPE cells also leads to upregulation of IL-1ra, shown to be highly expressed by RPE cells in response to IL-1β and TNFα stimulation [57]. Interestingly, IL-1β is highly expressed in iPSC-derived RPE cells carrying the AMD risk variant *CFH* 402H [42], and TNFα accumulates in the BrM and choroid in eyes from *CFH* 402H donors [58].

Our results are in line with independent observations that in AMD, as well as in the presence of the *CFH* 402H variant, inflammation is increased. However, the signaling pathways involved in regulating inflammation in RPE cells are not fully characterized, and most importantly, the signaling pathways modulated by FH, not only in RPE cells, are not well known. Recently, based on the phenotype of iPS-RPE cells carrying the *CFH* Y402H polymorphism, it has been speculated that the complement system and the NF-κB pathway may be part of complex crosstalk of signaling pathways with the scope of regulating RPE cell homeostasis [43].

Interestingly, most cytokines differentially regulated in FH-deprived conditions are, indeed, targets of the NF-κB pathway. We have proven here, that NF-κB activation follows suppression of *CFH* expression, which in turn results in an upregulation of NF-κB dependent cytokines. Reducing NF-κB levels lead to a reduction in the expression and secretion of most of the upregulated cytokines, including IL-6, IL-8, CCL2, Serpin E1, GM-CSF, and CXCL1/GROα. Consequently, the cytokine profile of the siCFH + siRELA group clusters tightly with the siNeg control. NF-κB consists of transcription factor complexes expressed in most cell types and can be activated in response to various stimuli or stressors, which allow the cell to respond and adapt to variations in the microenvironment, including complement activation levels and oxidative stress [32].

In many cell types and pathologies, the NF-κB is suggested to play a prosurvival role in the context of cell response to complement-mediated damage. Thus, mouse fibroblasts, HELA cells, and HEK293 cells lacking NF-κB p65, are all more sensitive to complement-mediated damage [59]. Moreover, HUVEC cells and human coronary endothelial cells (ECs) show NF-κB activation in response to MAC formation [60]. Data from in vitro and in vivo models of Alzheimer disease (AD) show that astroglia, as the principal site of NF-ƙB overactivation in the brain, is responsible for the NF-κB-dependent increase in C3. Importantly in this study, NF-κB binding sites were confirmed in the *C3* promoter [61]. Moreover, results from the kidney in vivo models of renal injury provide evidence that the NF-κB pathway plays an important role in renal damage mediated by enhanced local complement activation [62].

However, it is important to note that RPE cells, in contrast to all these previously mentioned cell types, show a significant level of tolerance to complement-mediated damage [63]. This may explain why they do not exploit the NF-κB pathway to respond to external complement stimulation, but rather regulate this pathway to maintain physiological levels of complement and inflammatory factors. Here in our model, the NF-κB pathway was not seen activated as a prosurvival pathway to respond to a complement activation mediated insult, since the addition of neither FH, C3, nor C3b had an impact on NF-κB activity, suggesting a possible intracellular function of FH in RPE cells. Consistent with our findings, FH silencing in clear cell renal cell carcinoma (ccRCC) and lung cancer also led to NF-κB activation, independent from extracellular complement sources [64].

Another major stimulus for NF-κB activation is oxidative stress, which has also been reported for RPE cells previously. For instance, ARPE19 cells show increased phosphorylation in NF-κB p65 in response to either short or long exposure to H_2_O_2_ [65]. In our previous study, we showed that FH loss increases oxidative and metabolic stress, and both stressors can induce NF-κB activation as a survival mechanism. We have also shown that genes involved in the oxidative stress response and in mitochondrial stability (e.g., PGC1α) were differentially regulated by complement activation—interestingly, these are also targets of the NF-κB pathway.

The observation, that a subset of cytokines (MIP-1α, MIP-1β, and IL-1ra), which were increased upon FH loss, were not reduced after silencing of NF-κB p65, supports the hypothesis that multiple pathways are likely involved in the interplay between complement system regulation and cell balance. Several pathways have been described as being involved in the homeostasis of RPE cells, which could be regulated by FH.

For example, deficiency of *CXCR5* causes defects in RPE cells and an AMD-like phenotype in *CXCR5* knockout mice, and transcriptome analysis of primary RPE cells from these mice highlights the PI3K-Akt and mTOR signaling pathways as important for RPE homeostasis [66]. Another possibility involves regulating the transcription factor AP1, which is regulated together with NF-κB in ARPE-19 cells in response to blue-light mediated damage [67].

Further studies will be necessary to understand the mechanism by which FH (and/or FHL-1) participates in the signaling network governing RPE cell balance. It is still debatable whether intracellular FH plays an important role in RPE cells, in the same way as in T-cells, where it modulates mTOR and T-cell activation [68]. Indeed, being co-expressed in RPE cells, FH could directly modulate NF-κB pathway activation on the protein level. Alternatively, the NF-κB pathway could be activated as part of the oxidative stress response in RPE cells in the absence of FH as an antioxidant factor.

Although the exact mechanism remains to be discovered, our data contribute to the understanding of how risk alleles in *CFH*, which result in reduced FH/FHL-1 activity, may contribute to excessive inflammation, as part of AMD pathology. We suggest that in RPE cells, FH and the NF-κB pathway are part of a complex network of signaling pathways, responsible for maintaining cellular homeostasis. In case either one of them is dysregulated, due to genetic risk, age, and/or local stressors, the RPE microenvironment changes towards a proinflammatory AMD-like phenotype, with NF-κB, as well as the alternative complement pathway acting as major protagonists.

## 4. Materials and Methods

### 4.1. Cell Culture and Experimental Settings

The human RPE cell line hTERT-RPE1 and ARPE19 were obtained from the American Type Culture Collection (ATCC). Cells were maintained in Dulbecco’s modified Eagle’s medium (DMEM; Gibco, Waltham, MA, USA) containing 10% fetal calf serum (FCS; Gibco, Waltham, MA, USA), penicillin (100 U/mL), streptomycin (100 µg/mL) in a humidified atmosphere containing 5% CO_2_. Cells were seeded in a complete growth medium without phenol red in 12-well plates and were attached overnight. Gene silencing was performed with Viromer Blue reagent according to the manufacturer’s instructions (Lipocalyx, Halle, Germany). Culture medium was substituted with fresh medium, and siRNA mixture was added dropwise. We employed a mix of three different double-strand hairpin interference RNAs specific for either *CFH* or *RELA* and a negative control (Neg), recommended by the provider (IDT technologies, Belgium). In experiments where double silencing was required (siCFH + siRELA), an additional amount of siNeg siRNA was added in the single silencing samples (siNeg, siRELA, and siCFH) to keep equal concentrations. Cells were then maintained in a serum-free medium for the indicated time, and where indicated, the medium was supplemented with FH (1 µg/mL), C3 (0.1 µg/mL), or C3b (0.1 µg/mL) (CompTech, Tyler, TX, USA).

### 4.2. RNA Extraction, cDNA Synthesis, and Quantitative RT-PCR

At the indicated time points, total RNA was extracted with PureZOL reagent, according to the manufacturer’s instructions (Bio-Rad Laboratories, Des Plaines, IL, USA), and cDNA was synthesized via reverse-transcription of 1 μg of RNA using M-MLV Reverse Transcriptase (Promega, Madison, WI, USA). cDNA was used to analyze differences in gene expression by qRT-PCR employing iTaq Universal SYBR Green Supermix (Bio-Rad Laboratories, Des Plaines, IL, USA) along with gene-specific forward and reverse primers (10 μM) listed in Table 1. PCR protocol includes 40 cycles of: 95 °C (5 s) and 57 °C (30 s), carried on CFX96 Real-Time System (Bio-Rad Laboratories, Des Plaines, IL, USA). Relative mRNA expression of each gene of interest (GOI, Table 1) was quantified using 60 s acidic ribosomal protein P0 (PRLP0) as the housekeeping control gene.

### 4.3. Western Blotting

Protein expression was analyzed in both cell lysates and cell supernatants. After debris removal, cell culture supernatants were precipitated with ice-cold acetone. For protein analysis of cell lysates, cells were lysed in Pierce IP Lysis Buffer, containing Halt Protease and Phosphatase Inhibitor (Thermo Fisher, MA, USA). Protein concentrations were determined with the Bradford quantification assay, using BSA as a standard. Equal amounts of cell lysates or equal volumes of cell supernatants were prepared in NuPAGE LDS Sample Buffer, containing reducing agent (Thermo Fisher, Boston, MA, USA) and analyzed on Novex 8–16% Tris-Glycine gels (Invitrogen, Waltham, MA, USA). Subsequently, proteins were transferred onto PVFD membranes, and Western blot detection was carried out as previously described [31], using the primary antibodies listed in Table 2. Pictures were acquired with a FusionFX imaging system (Vilber Lourmat, Collégien, France), and the intensity density of individual bands was quantified using the ImageJ software (Version 1.53a).

### 4.4. C3b ELISA

C3/C3b ELISA to evaluate the concentration of C3/C3b in cell culture supernatants was performed according to the manufacturer’s instructions (Abcam, UK). Samples and standard controls were loaded in 96 well-plates coated with specific C3b antibodies. Absorbance was read at a wavelength of 450 nm immediately after the assay procedure using a Spark multimode microplate reader (Tecan, Männedorf, Switzerland). Subtraction readings at 570 nm were taken to correct optical imperfections.

### 4.5. Cytokine Array

The Proteome Profiler Human Cytokine Array Kit (R&D Systems, McKinley Pl, MN, USA) was employed to determine the relative levels of 36 different cytokines, and the assay was performed according to the manufacturer’s instructions. Briefly, the membranes were blocked for 1 h at room temperature. The cellular supernatant samples were further prepared by mixing 400 μL of the sample with 500 μL Array Buffer 4, 600 μL Array Buffer 5, and 15 μL of Detection Antibody Cocktail and incubated for 1 h at room temperature. The prepared mixture was added to the membranes, followed by an incubation period overnight at 4 °C. The membranes were washed 3 × 10 min, incubated in diluted HRP-Streptavidin (1:2000) for 30 min, and washed again for 3 × 10 min. The Chemi Reagent Mix (1:1 ratio) was dropped onto the membranes, incubated for 1 min, and the signal was detected by FusionFX (Vilber Lourmat, Lamirault, Collégien) in the automatic mode, and additionally, in an individual programmed mode with an increasing detection time of: 0.5, 1, 1.5, 2, 4, 6, 10 min. The results were evaluated with the Fusion software and ImageJ by measuring the intensity density.

### 4.6. Bioinformatic Analyses

Data analysis of the raw values from the cytokine array, as obtained and measured using ImageJ, were normalized to the positive control. For principal component analysis (PCA), values were Pareto scaled by dividing each variable by the square root of the standard deviation to minimize the effect of small noisy variables.

The Variable Importance in Projection (VIP) in a Partial Least Square Discriminant Analysis (PLS-DA) was used to identify the most discriminative cytokines for each biological group following siRNA treatment. Similar to the PCA analysis and normalized to the positive control, Pareto scaled values were used. PCA and VIP score analysis were carried out using the R package MetaboAnalystR, integrated into the publicly available platform for statistical analysis metaboanalyst.ca [69].

### 4.7. Statistical Analysis

The data are presented as mean with the standard error of the mean (SEM) and were generated and tested for their significance with GraphPad Prism 8 software, version 8.4.3. All data sets were tested for normal distribution, assessed with the Shapiro-Wilk normality test. Depending on the normal distribution and the parameters to be compared, the following tests were performed: A Mann-Whitney test was used in case of non-normal distribution, an unpaired Student´s *t*-test was used to compare siNeg vs. either siCFH or siRELA condition and to compare siCFH vs. siCFH + siRELA. A ratio paired *t*-test was used to compare the relative changes between siCFH vs. siCFH+siRELA and siCFH vs. siCFH treated (FH, C3 or C3b), only when both conditions were normalized to siNeg control. Values were considered significant with *p* < 0.05.

## Figures and Tables

**Table 1:** List of primers used in this study.

Target Gene	Forward Primer	Reverse Primer
*CFH*	5′-CTG ATC GCA AGA AAG ACC AGT A 3′	5′-TGG TAG CAC TGA ACG GAA TTA G 3′
*CFB*	5′-GCT GTG AGA GAG ATG CTC AAT A 3′	5′-GAC TCA CTC CAG TAC AAA G 3′
*C3*	5′-ACG GCC TTT GTT CTC ATC TC 3′	5′-CAA GGA AGT CTC CTG CTT TAG T 3′
*C5*	5′-CGA TGG AGC CTG CGT TAA TA 3′	5′-CTT GCG ACG ACA CAA CAT TC 3′
*CFI*	5′-TAC TCA CCT CTC CTG CGA TAA 3′	5′-GGG CAC TGA TAC GGT AGT TTA C 3′
*CCL2*	5′-GGC TGA GAC TAA CCC AGA AAC 3′	5′-GAA TGA AGG TGG CTG CTA TGA 3′
*IL6*	5′-CCA GGA GAA GAT TCC AAA GAT GTA 3′	5′-CGT CGA GGA TGT ACC GAA TTT 3′
*CXCL8*	5′-AAA TCT GGC AAC CCT AGT CTG 3′	5′-GTG AGG TAA GAT GGT GGC TAA T 3′
*RELA*	5′-CTG TCC TTT CTC ATC CCA TCT T 3′	5′-TCC TCT TTC TGC ACC TTG TC 3′
*PRLP0*	5′-GGA GAA ACT GCT GCC TCA TAT C 3′	5′-CAG CAG CTG GCA CCT TAT T 3′

**Table 2:** List of primary antibodies used in this study.

Antibody	Supplier	Number
β-actin	Cell Signaling	#3700
Complement C3	Invitrogen, ThermoFisher	#PA5-21349
Factor H (FH)	SantaCruz Biotechnology	sc-166608
p-NF-κB p65	Cell Signaling	#3033
Total NF-κB p65	Cell Signaling	#8242

## Data Availability

Not Applicable.

## Supplementary Materials

The following are available online at www.mdpi.com/article/10.3390/ijms22168727/s1, Figure S1: FH reduction leads to increased expression levels of C3 and inflammatory cytokines. Figure S2: RPE cells deprived of FH show NF-κB activation. Figure S3: Impact of exogenous FH, C3, and C3b in hTERT-RPE1 cells. Figure S4. Cytokines profile in hTERT-RPE1 cells. Figure S5. Impact of exogenous FH, C3, and C3b in hTERT-RPE1 cells.
