# Supplementary material for: CFH Loss in Human RPE Cells Leads to Inflammation and Complement System Dysregulation via the NF-κB Pathway
Source: Int J Mol Sci. 2021 Aug 13;22(16):8727. doi: 10.3390/ijms22168727 (PMC8396051; doi:10.3390/ijms22168727)
Supplement: Supplementary file 1 [file ijms-22-08727-s001.zip › ijms-1285367-supplementary.pdf]

International Journal of *Molecular Sciences*

MDPI

Article

Supplementary Material: CFH loss in human RPE cells leads to inflammation and complement system dysregulation *via* the NF-κB pathway

Angela Armento[1]*, Tiziana L. Schmidt[1]*, Inga Sonntag[1], David Merle[1], Mohamed Ali Jarboui[1], Ellen Kilger[1], Simon J. Clark[1,2,3]; Marius Ueffing[1,2]

[1] Institute for Ophthalmic Research, Department for Ophthalmology, Eberhard Karls University of Tübingen, Tübingen, Baden-Württemberg, 72076, Germany
angela.armento@uni-tuebingen.de (A.A), TizianaLuisa@web.de (T.L.S), inga.Sonntag@uni-tuebingen.de (I.S), david.merle@medunigraz.at (D.M), mohamed-ali.jarboui@uni-tuebingen.de (M.A.J), ellen.kilger@uni-tuebingen.de (E.K.), marius.ueffing@uni-tuebingen.de (M.Ue)

[2] University Eye Clinic, Department for Ophthalmology, Eberhard Karls University of Tübingen, Tübingen, Baden-Württemberg, 72076, Germany
simon.clark@uni-tuebingen.de (S.J.C)

[3] Lydia Becker Institute of Immunology and Inflammation, Faculty of Biology, Medicine and Health, University of Manchester, Manchester, M13 9PT, UK

* Both authors share equal first authorship (A.A., T.L.S); correspondence should be addressed to:
Angela Armento, PhD, angela.armento@uni-tuebingen.de, Phone: +49 7071 29 84953; Marius Ueffing, PhD, marius.ueffing@uni-tuebingen.de. Institute for Ophthalmic Research, Department for Ophthalmology, Eberhard Karls University of Tübingen, Tübingen, Baden-Württemberg, 72076, Germany.

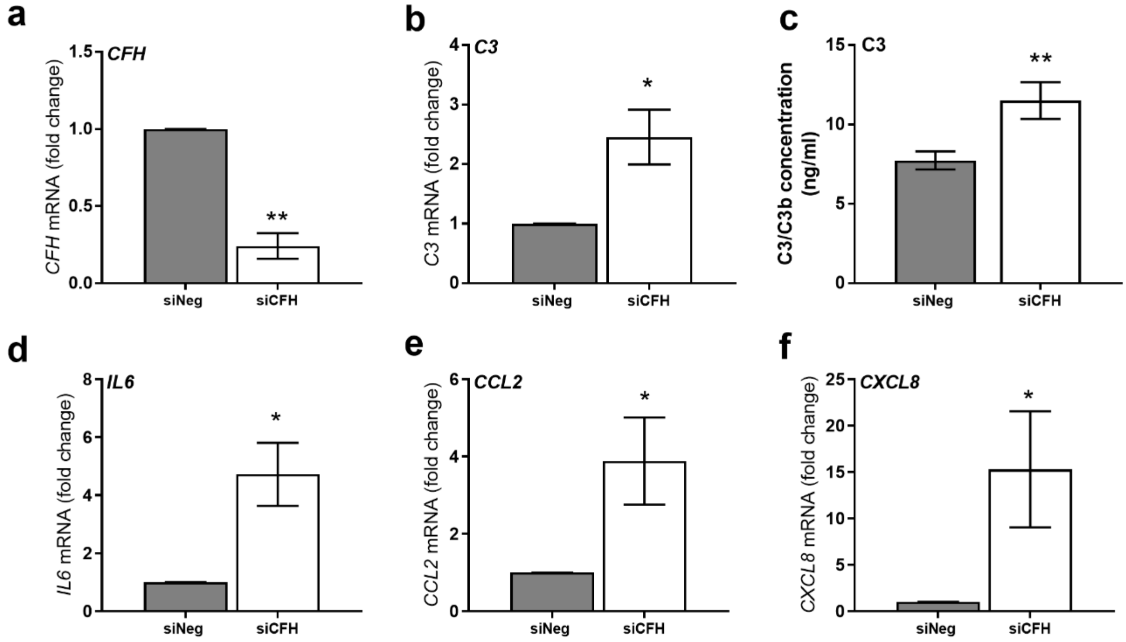

Figure S1. FH reduction leads to increased expression levels of C3 and inflammatory cytokines. a Evaluation of *CFH* expression by qRT-PCR analyses. **b** Monitoring of complement component 3 (*C3*) gene expression by qRT-PCR analyses in ARPE19 cells. **c** C3/C3b ELISA analyses of cell culture supernatants of ARPE19 cells. SEM is shown, n=4. **d-f** monitoring of gene expression of **d**

interleukin-6 (*IL6*), **e** C-C Motif Chemokine Ligand 2 (*CCL2*) and **f** interleukin-8 (*CXCL8*). qRT-PCR data are normalized to housekeeping gene *PRPL0* using Δ ΔCt method. SEM is shown, n=4. Significance was assessed by Student's t-test. *$p<0.05$, **$p<0.01$.

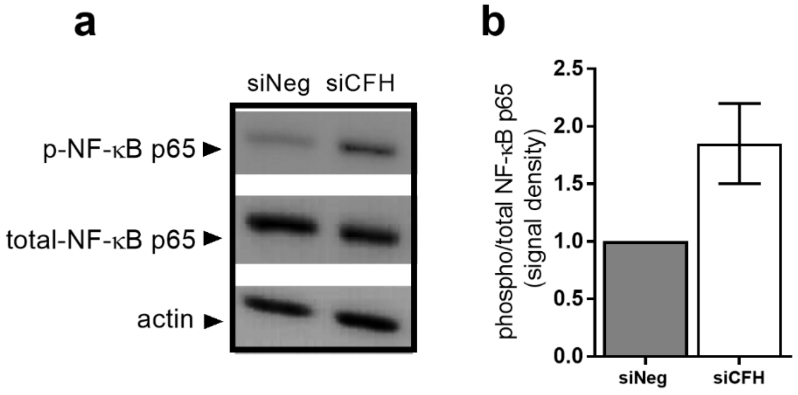

Figure S2. RPE cells deprived of FH show NF-κB activation. **a** Western blot analyses of phosphorylated and total levels of NF-κB p65 subunit in cell lysates of ARPE19 cells. Total actin was used as loading control**. b** Quantification of signal density of at least 3 independent experiments as reported in **a.** Bars indicate the signal density ratio between levels of phosphorylated and total NF-κB p65 subunit. Western Blot images were cropped, and full-length blots are provided in Supplementary Material.

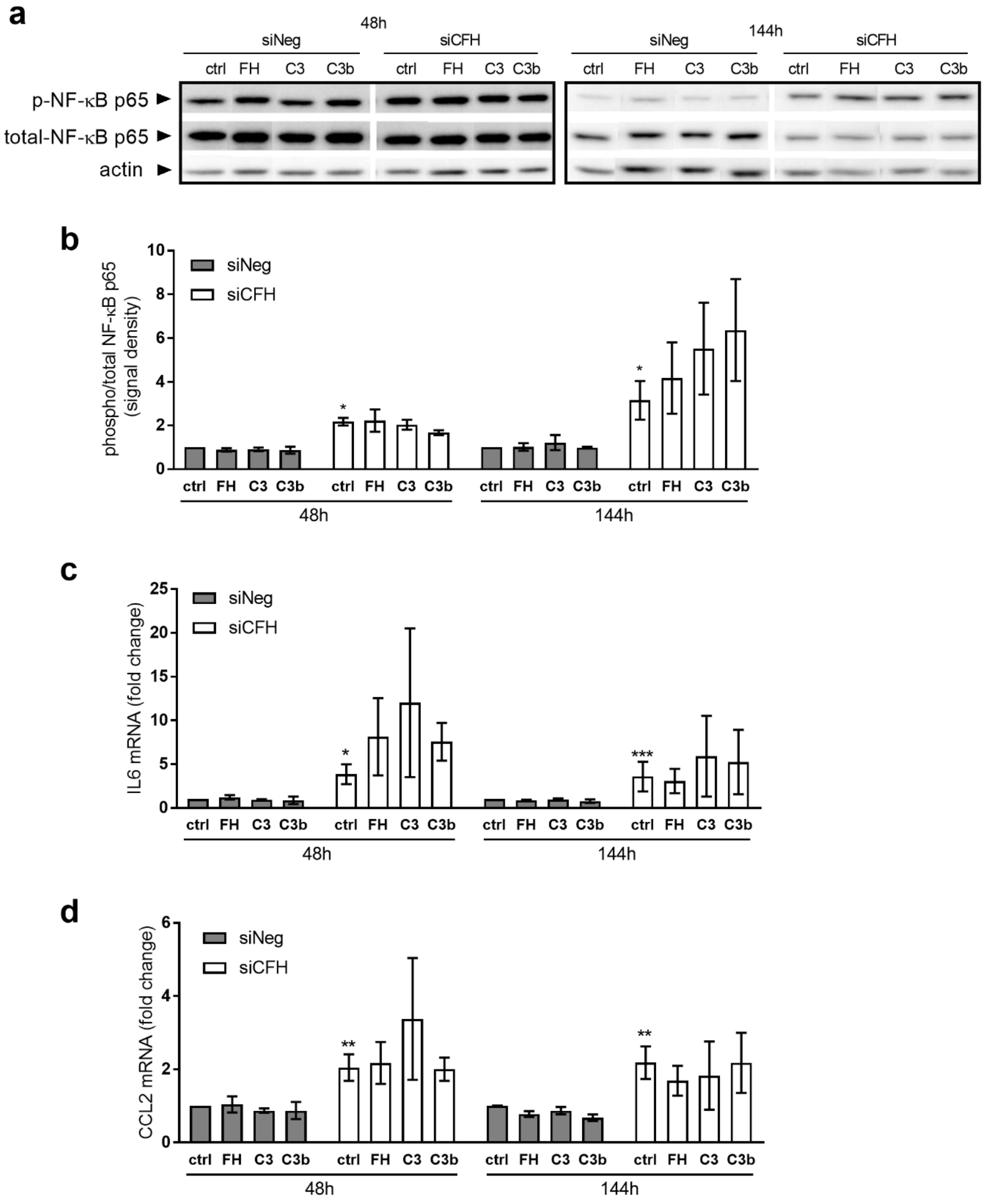

Figure S3. Impact of exogenous FH, C3 and C3b in hTERT-RPE1 cells. a Western blot analyses of phosphorylated and total levels of NF-κB p65 subunit in in cell lysates of hTERT-RPE1. Total actin was used as loading control **b** Quantification of signal density of at least 4 independent experiments in the conditions reported in a. Bars indicate the signal density ratio between phosphorylated and total levels of NF-κB p65 subunit. **c-d** gene expression analyses of hTERT-RPE1 cells in the conditions reported in a: *IL6* in panel c, *CCL2* in panel d. SEM is shown, n=4-8. Western Blot images were cropped, and full-length blots are provided in Supplementary Material. Significance was assessed by Student's t-test. *p<0.05, **p<0.01, *** p<0.001.

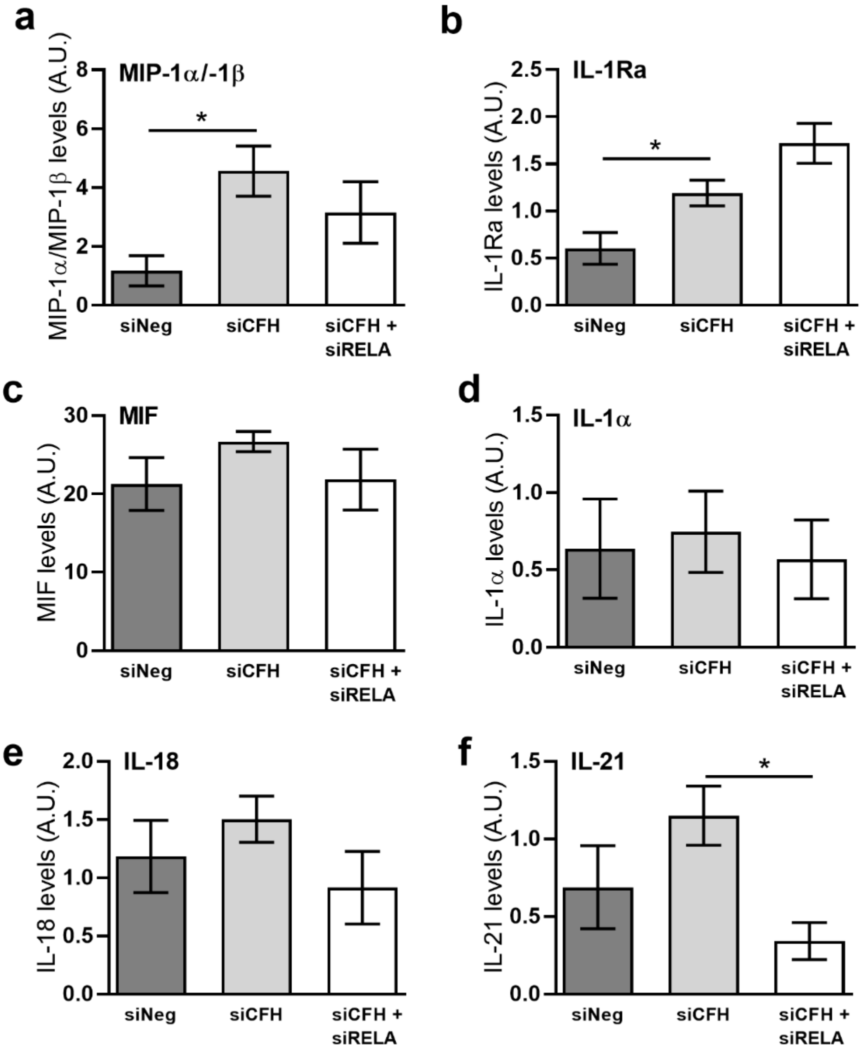

Figure S4. Cytokines profile in hTERT-RPE1 cells.. **a-f** Quantification of signal density in the conditions reported in Fig. 3a: **a** C-C motif chemokine ligand 3/4, MIP-1α/-1β; **b** interleukin 1 receptor antagonist, IL-1Ra. **c** macrophage migration inhibitory factor, MIF; **d** interleukin-1 alpha, IL-1a; **e** interleukin-18, IL-18; **f** interleukin-21, IL-21. SEM is shown. n=3. Significance was assessed by Student's t-test. *p<0.05.

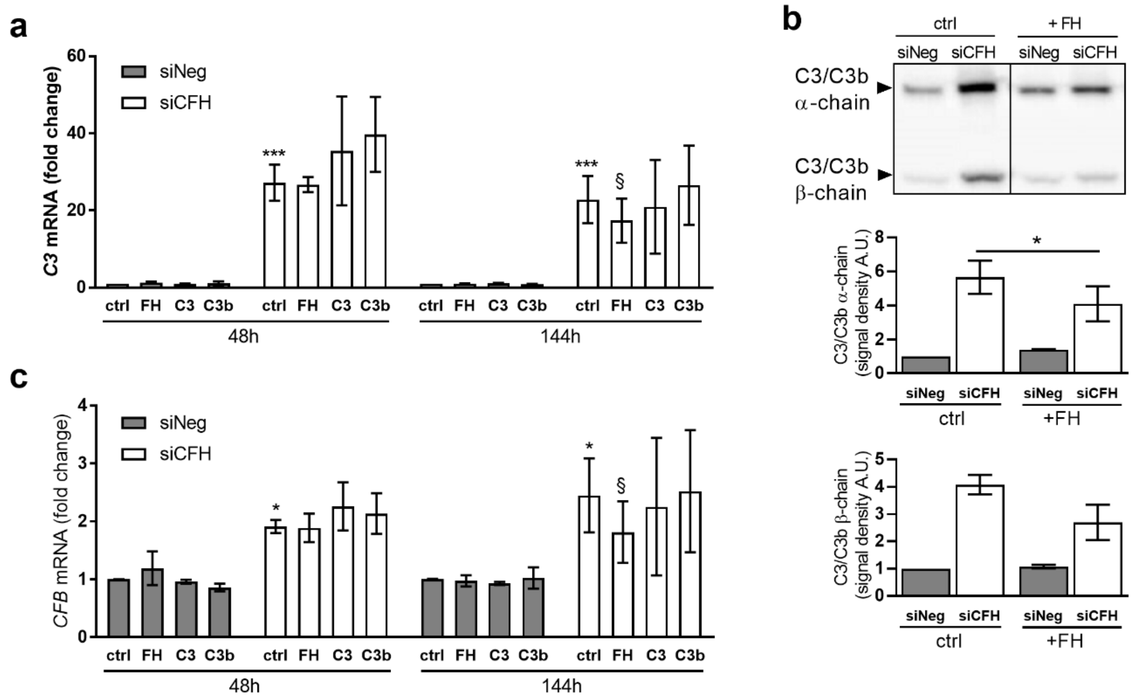

Figure S5. Impact of exogenous FH, C3 and C3b in hTERT-RPE1 cells. a *C3* gene expression analyses of hTERT-RPE1 cells **b** Western blot analyses and quantification of phosphorylated and total levels of NF-κB p65 subunit in in cell lysates of hTERT-RPE1. Total actin was used as loading control. Western Blot images were cropped, and full-length blots are provided in Supplementary Material **d**. **c** *CFB* gene expression analyses of hTERT-RPE1 cells.

SEM is shown, n=4-8. Significance was assessed by Student's t-test. Comparison between siNeg and siCFH: *p<0.05, **p<0.01, *** p<0.001. Comparison between siCFH ctrl and siCFH FH: § p <0.05.
